# Multidrug Resistant 2009 A/H1N1 Influenza Clinical Isolate with a Neuraminidase I223R Mutation Retains Its Virulence and Transmissibility in Ferrets

**DOI:** 10.1371/journal.ppat.1002276

**Published:** 2011-09-29

**Authors:** Erhard van der Vries, Edwin J. Veldhuis Kroeze, Koert J. Stittelaar, Martin Linster, Anne Van der Linden, Eefje J. A. Schrauwen, Lonneke M. Leijten, Geert van Amerongen, Martin Schutten, Thijs Kuiken, Albert D. M. E. Osterhaus, Ron A. M. Fouchier, Charles A. B. Boucher, Sander Herfst

**Affiliations:** 1 Erasmus Medical Centre, Department of Virology, Rotterdam, The Netherlands; 2 Viroclinics Biosciences BV, Rotterdam, The Netherlands; Johns Hopkins University - , Bloomberg School of Public Health, United States of America

## Abstract

Only two classes of antiviral drugs, neuraminidase inhibitors and adamantanes, are approved for prophylaxis and therapy against influenza virus infections. A major concern is that influenza virus becomes resistant to these antiviral drugs and spreads in the human population. The 2009 pandemic A/H1N1 influenza virus is naturally resistant to adamantanes. Recently a novel neuraminidase I223R mutation was identified in an A/H1N1 virus showing cross-resistance to the neuraminidase inhibitors oseltamivir, zanamivir and peramivir. However, the ability of this virus to cause disease and spread in the human population is unknown. Therefore, this clinical isolate (NL/2631-R223) was compared with a well-characterized reference virus (NL/602). *In vitro* experiments showed that NL/2631-I223R replicated as well as NL/602 in MDCK cells. In a ferret pathogenesis model, body weight loss was similar in animals inoculated with NL/2631-R223 or NL/602. In addition, pulmonary lesions were similar at day 4 post inoculation. However, at day 7 post inoculation, NL/2631-R223 caused milder pulmonary lesions and degree of alveolitis than NL/602. This indicated that the mutant virus was less pathogenic. Both NL/2631-R223 and a recombinant virus with a single I223R change (recNL/602-I223R), transmitted among ferrets by aerosols, despite observed attenuation of recNL/602-I223R *in vitro*. In conclusion, the I223R mutated virus isolate has comparable replicative ability and transmissibility, but lower pathogenicity than the reference virus based on these *in vivo* studies. This implies that the 2009 pandemic influenza A/H1N1 virus subtype with an isoleucine to arginine change at position 223 in the neuraminidase has the potential to spread in the human population. It is important to be vigilant for this mutation in influenza surveillance and to continue efforts to increase the arsenal of antiviral drugs to combat influenza.

## Author Summary

Recently, a 2009 pandemic A/H1N1 influenza virus was isolated from an immune compromised patient, with antiviral resistance to the neuraminidase inhibitor class of drugs. This virus had an amino acid change in the viral neuraminidase enzyme; an isoleucine at position 223 was substituted for an arginine (I223R). Patients infected with a pandemic virus that is resistant to all neuraminidase inhibitors, would leave physicians without antiviral treatment options, since these viruses are naturally resistant to the other class of antivirals, the adamantanes. To date, it is unknown if this I223R mutant virus is affected in its ability to cause severe disease and to transmit to other humans. Therefore, we have addressed this question by comparing the I223R mutant virus with a wild type reference virus in a ferret pathogenicity and transmission model. We found that the I223R mutant virus was not severely affected in its pathogenicity, although fewer lung lesions and alveolitis scores were found for the I223R mutant virus. In addition, we demonstrated that this virus transmitted efficiently to naïve ferrets. Consequently, we conclude that this I223R mutant virus has the potential to cause disease and may spread among humans. Therefore, influenza surveillance for this resistance pattern is advised.

## Introduction

Two classes of antiviral drugs are approved for prophylaxis and therapy of influenza virus infected patients [1]. Antiviral therapy against the new (swine-origin) 2009 pandemic A/H1N1 influenza virus relies on the neuraminidase inhibitor (NAI) class of antiviral drugs only, because this subtype is resistant to the adamantane class (amantadine and rimantadine) of drugs [2]. In 2009 pandemic influenza viruses, this resistance pattern is mainly caused by an asparagine at amino acid position 31 (N31) in the viral M2 membrane protein. Fortunately, NAI treatment, both as prophylaxis and therapy, has been shown to be effective against most 2009 pandemic H1N1 virus infections so far [3], [4].

To date, the incidence of NAI resistant 2009 pandemic A/H1N1 viruses is very low. Nevertheless, 565 cases of patients infected with an (H275Y, N1 numbering) oseltamivir (OS) resistant virus have been reported to the World Health Organization [5]. In most of these cases, OS resistance was found in patients receiving prolonged antiviral therapy, in particular patients under immunosuppressive therapy [6]. The H275Y mutant viruses are cross-resistant to peramivir (PER), but remain susceptible to zanamivir (ZA). Successful clearance of a H275Y mutant virus from a patient treated with ZA was reported previously [7].

Within the first years after approval of the NAIs in 1999, antiviral resistance in influenza viruses at a population level was rare (0.4%). In clinical trials, the incidence of resistant viruses was higher, varying from 0.4 to 1% in adults and up to 18% in young children [8], [9]. However, a dramatic increase, up to 100%, of de novo circulating oseltamivir-resistant A/H1N1 viruses characterized the epidemic seasons of 2007-2008 and 2008-2009 [10], [11]. This resistance phenotype was also caused by a H275Y mutation. Remarkably, earlier studies on H275Y mutant H1N1 viruses had characterized these viruses as attenuated and not of clinical importance [12], [13], [14]. The resistant viruses from 2007-2008 did not seem to be affected in replication capacity, transmissibility and their ability to cause severe disease in humans [15], [16], [17]. A compensatory role was assigned to the NA amino acid changes V234M, R222Q and D344N [18], [19]. These substitutions may have restored the initial loss of NA activity due to the NAI resistance mutation and facilitated the appearance of the H275Y change in the epidemic influenza A/H1N1 viruses that circulated before the 2009 outbreak of the new pandemic virus. Recently, several research groups have studied the fitness of H275Y mutant pandemic influenza A/H1N1 viruses using both *in vitro* and *in vivo* experiments [20], [21], [22], [23], [24]. Overall, these data indicate that pandemic viruses with the NA H275Y substitution were comparable to their oseltamivir susceptible counterparts in pathogenicity and transmissibility in animal models.

Recently, the identification of a novel multidrug resistant 2009 pandemic A/H1N1 virus was reported, isolated from an immune compromised child with reduced susceptibility to all NAIs [25]. An isoleucine to arginine substitution at position 223 in NA (I223R, N1 numbering) was detected in the patient after antiviral therapy with OS had failed due to the emergence of the H275Y mutation and therapy was switched to ZA. This I223R containing isolate, in which the H275Y mutation had disappeared, showed reduced susceptibility to OS (45-fold), PER (7-fold) and ZA (10-fold). *In vitro* analysis showed that reversion of the arginine to isoleucine fully restored NAI susceptibility. In another case, an I223R/H275Y double mutant virus was isolated that showed high resistance to the NAIs [26]. In combination with the natural resistance of pandemic A/H1N1 viruses to adamantanes, an infection of such a multi-drug resistant virus leaves physicians without antiviral treatment options. The emergence of this pandemic 2009 A/H1N1 virus prompted us to investigate the properties of this clinical isolate by evaluating its *in vitro* replication kinetics and its pathogenicity and transmissibility in the ferret model. We here show that this 2009 pandemic influenza A/H1N1 clinical isolate, harboring a neuraminidase I223R substitution retains its virulence and transmissibility, but is less pathogenic than a virus prototype without this mutation. In addition, recombinant NL/602/09 with a single I223R amino acid substitution transmitted as well as its recombinant parental virus, suggesting that no additional mutations are needed to compensate for the presence of this I223R mutation in the 2009 pandemic A/H1N1 virus backbone.

## Results

### Sequence comparison of virus isolates

A pandemic 2009 influenza virus with reduced susceptibility to all NAIs that was isolated from a Dutch immune compromised child was studied here. Full genome sequencing of this clinical isolate A/NL/2631_1202/2010 (NL/2631-R223, GenBank accession numbers JF906180-906187) harboring an I223R mutation in the neuraminidase was performed. Since no drug susceptible virus had been isolated from this patient before start of antiviral therapy, the well-characterized NAI-susceptible virus isolate A/NL/602/2009 (NL/602, GenBank accession numbers CY046940-046945 and CY039527-039528) was used as a reference virus in all experiments. This reference virus is a representative of pandemic H1N1 viruses that circulated in 2009, with only amino acid changes I108V and V407I (N1 numbering) in NA being unusual among the deposited sequences in the Influenza Research Database [27], [28]. Pair-wise comparison revealed, in addition to the amino acid change I223R, 5 amino acid differences in NA (V106I, V108I, N248D, N386D and I407V) and 1 in HA (S203T). The NA and HA amino acid positions are given according to the N1 and H1 numbering. Eleven additional amino acid differences were found in gene segments PB2 (3), PB1 (2), PA (2), NP (3) and NS (1) compared to NL/602. None of these mutations have previously been identified as a virulence marker or as a compensatory mutation involved in restoration of NA activity loss, as a result of the presence of resistance mutations. By studying these isolates, a direct comparison could be made between a NAI susceptible and a novel I223R resistant virus, but such comparison does not address the impact of the single I223R mutation directly. Therefore, we introduced the I223R mutation in the recNL/602 backbone, resulting in the drug-resistant recNL602-I223R, to evaluate the impact of the single I223R mutation on virus replication, virus shedding from the upper respiratory tract and transmissibility in the ferret model.

### I223R harboring isolate is not attenuated *in vitro*

**Figure 1:**
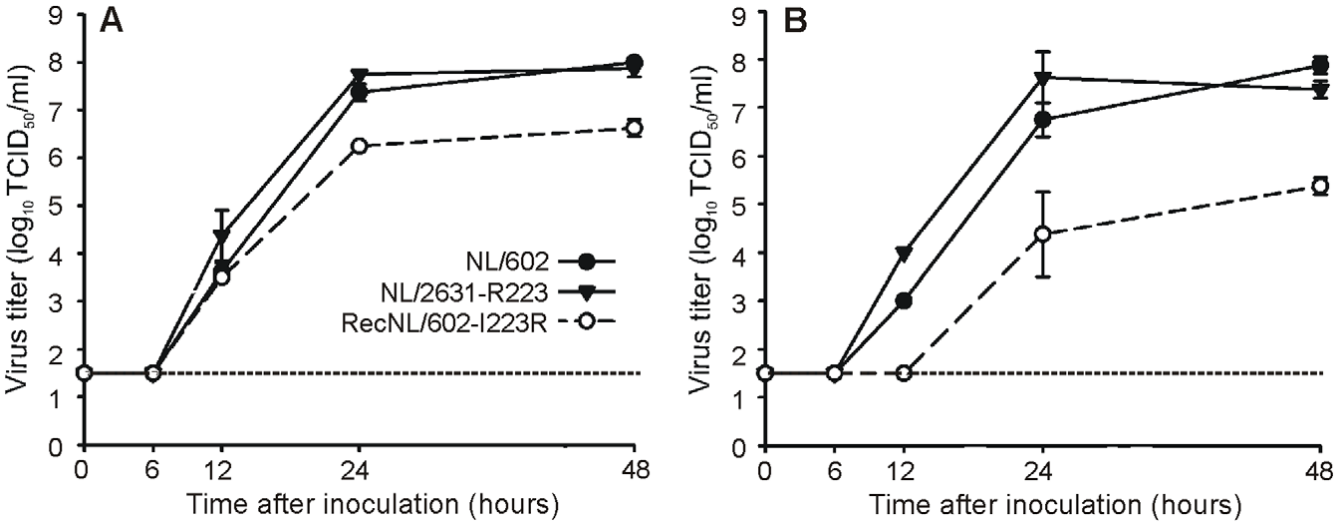
Replication kinetics in MDCK or MDCK-SIAT1 cells. MDCK (panel A) or MDCK-SIAT1 (panel B) cells were inoculated with 0.001 TCID_50_ virus per cell of recNL/602 (black circles), isolate NL/2631-R223 (black triangles) and recNL/602-I223R (open circles). Supernatants were harvested after 6, 12, 24, and 48 hours post infections and were titrated in MDCK cells. Geometric mean titers and standard deviations were calculated from two independent experiments. The lower limit of detection is indicated by the dotted line.

Virus replication was studied *in vitro* by multi-cycle replication kinetics of the viruses of interest. For this purpose, MDCK or MDCK-SIAT1 cell cultures were inoculated at a multiplicity of infection of 0.001 TCID_50_ per cell and at fixed time points supernatants were harvested to determine viral titers (Figure 1). Overall, the initial virus replication rates and end point titers were similar for the clinical isolate NL/2631-R223 and recNL/602.

A recombinant derivative of NL/602 with the I223R mutation in NA (recNL/602-I223R) replicated to lower peak titers in both cell lines compared to recNL/602 and NL/2631-R223. In addition, initial virus replication of recNL/602-I223R was delayed by 6 to 12 hours in MDCK-SIAT1 cells.

### No marked differences in virus replication in the respiratory tract of ferrets

**Figure 2:**
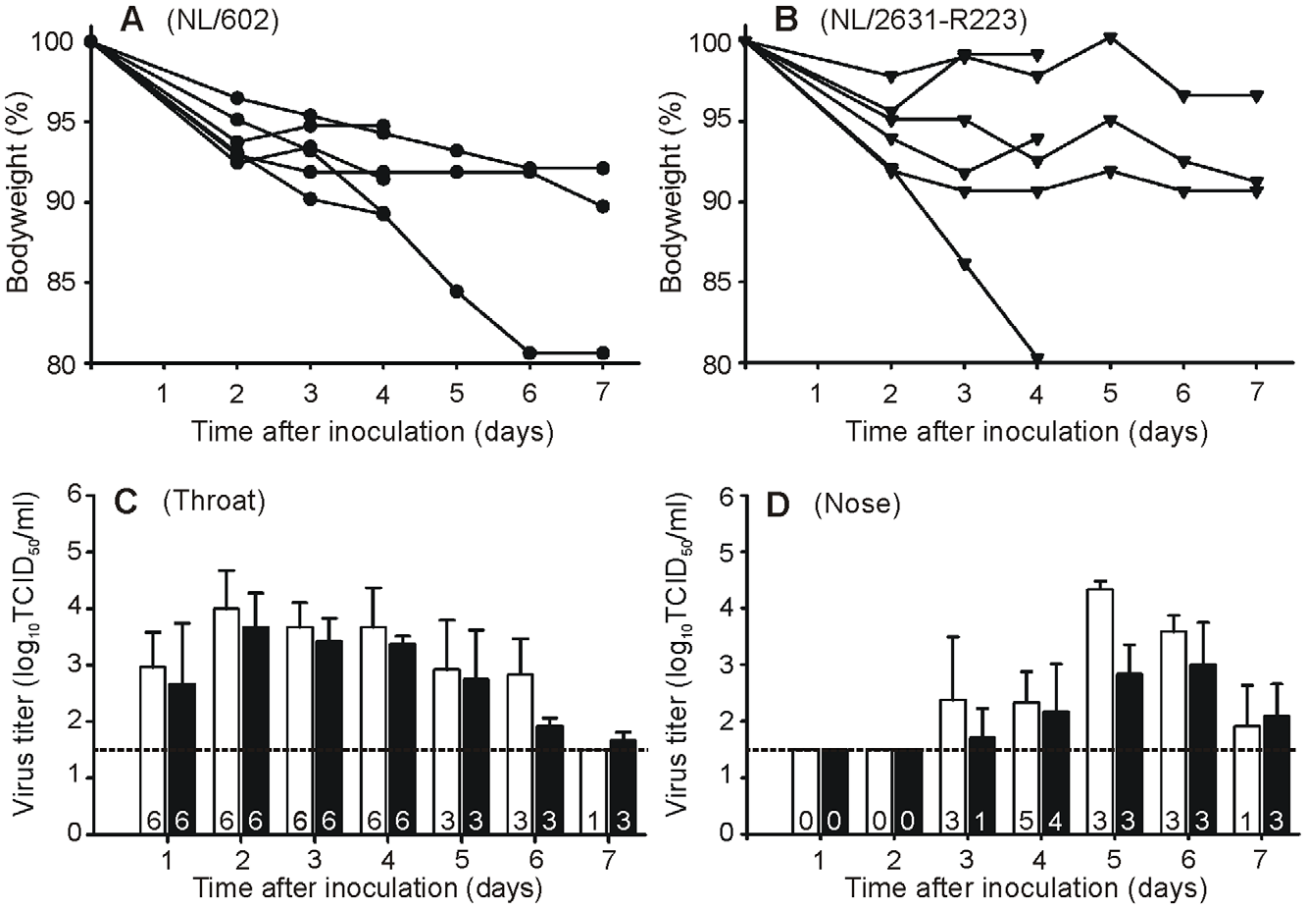
Ferret relative weight loss and virus shedding from the ferret upper respiratory tract. Ferrets were inoculated intratracheally with 1×10^6^ TCID_50_ of NL/602 or NL/2631-R223. Body weights for NL/602 (Panel A) and NL/2631-R223 (Panel B) inoculated animals are depicted as percentage of body weight relative to the time of inoculation. Data are shown for individual animals until the animals were euthanized at day 4 or 7 p.i.. Virus detection in throat (panel C) and nose swabs (panel D) is indicated for NL/602 (white bars), and NL/2631-R223 (black bars). Geometric mean titers from 6 (day 1 to 4) or 3 animals (day 5 to 7) are displayed and the error bars indicate the standard deviations. The number of influenza virus positive animals per day is depicted in each bar. The lower limit of detection is indicated by the dotted line.

The pathogenicity of clinical isolate NL/2631-R223 was compared with NL/602 in the ferret model that was previously established to study the ability of influenza viruses to cause pneumonia [29]. Two groups of 6 ferrets were inoculated intratracheally with 10^6^ TCID_50_ of virus. The animals were weighed daily as an indicator of disease. Over the 7-day period, no significant differences were observed in weight loss between the two groups inoculated with either virus. At day 4 post infection (p.i.), when there were still 6 animals present in each group, the mean percentage of weight loss was 8,2±2,4% and 7,6±6,7% for NL/602 and NL/2631-R223-inoculated animals respectively, not statistically significant (Figure 2A and B). In addition, no marked differences were observed for other clinical parameters, such as lethargy, sneezing and interest in food.

Nose and throat swabs were collected daily from the inoculated animals and virus titers were determined by end-point titration in MDCK cells. Infectious virus shedding from the throat was detected from day 1 p.i. onwards in all ferrets, with similar patterns of virus shedding from the throat of the animals in the two groups (Figure 2C). At day 4 p.i., 5 and 4 animals were shedding virus from the nose in the NL/602 and NL/2631-R223 inoculated group respectively (Figure 2D). Sequence analysis confirmed the presence of the I223R mutation in the respiratory samples collected at day 7 p.i. from the NL/2631-R223 inoculated ferrets.

**Figure 3:**
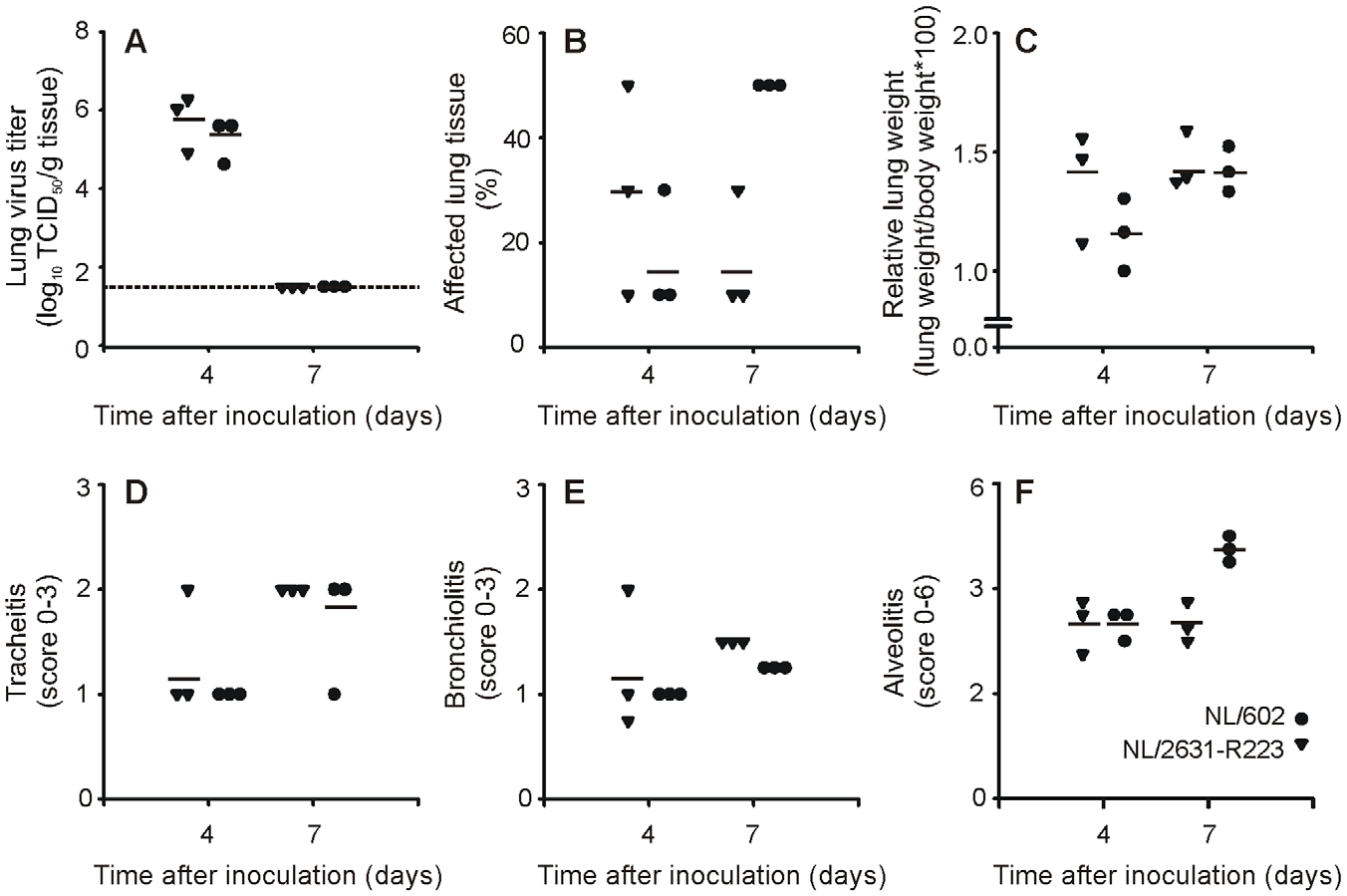
Semi-quantitative lung scores and histological examination of the infected ferret respiratory tract. Lung virus titers (panel A), percentage of affected lung tissue (panel B) and relative lung weights (panel C) were determined for lungs of ferrets inoculated with NL/2631-R223 (triangles) or NL/602 (circles) that were euthanized at day 4 or 7 p.i.. Semi-quantitative assessment of the extent and severity of the tracheitis (panel D), bronchiolitis (panel E) and alveolitis (panel F) are shown. Individual values are displayed. In panel A, the lower limit of detection is indicated by a dotted line.

At day 4 and 7 p.i., three animals of each group were euthanized and lungs were collected for virological and pathological examination. At day 4 p.i., no marked differences were found between the virus titers for both groups of ferrets (Figure 3A). At day 7 p.i., no virus was detected in the lungs of ferrets inoculated with either virus.

### Moderate pathogenicity of I223R harboring isolate

**Figure 4:**
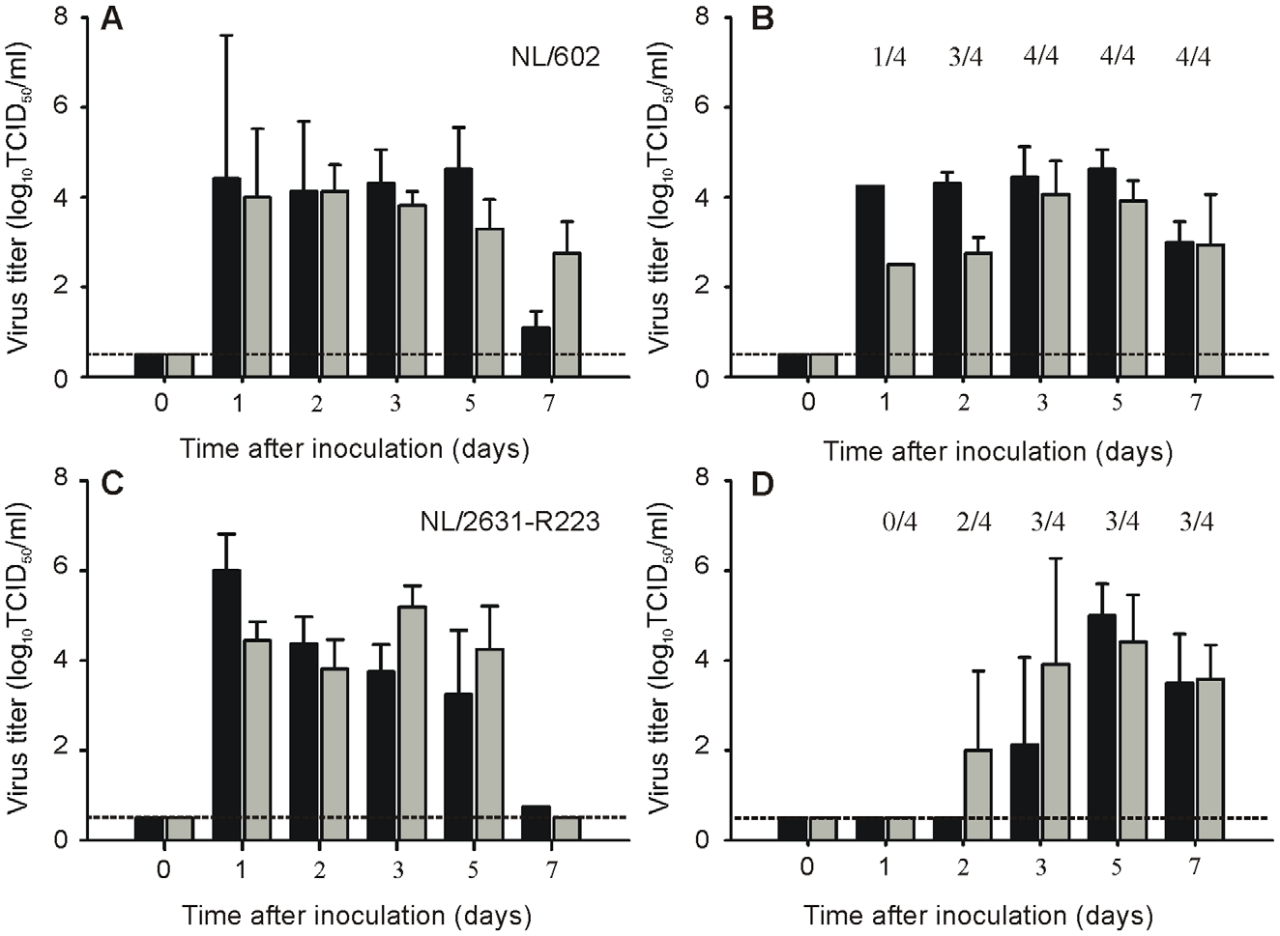
Transmission of NL/602 and NL/2631-R223 by aerosol or respiratory droplets in ferrets. Virus titers in throat (black bars) and nose swabs (grey bars) are displayed for inoculated (Panel A and C) and exposed ferrets (Panel B and D). The geometric mean titers of positive samples are displayed and the error bars indicate the standard deviations. The number of positive exposed animals per day is depicted. The lower limit of detection is indicated by the dotted line.

Gross pathology of the lungs of all animals revealed pulmonary lesions at day 4 and 7 p.i. (Figure 3B). At day 4 p.i., no marked difference was observed between the groups, but at day 7 p.i., the percentage of affected lung tissue was higher in the group inoculated with NL/602. The mean relative lung weight increased from day 4 to day 7, with no difference between the animals inoculated with NL/602 or NL/2631-R223 (Figure 3C). Histopathological examination of the lungs showed multifocal to coalescing alveolar damage in both groups characterized by the presence of macrophages and neutrophils within the lumina and thickened alveolar walls. At day 4 p.i., the severity of alveolitis did not differ between the two groups (Figure 4D). However, in agreement with the increased percentage of affected lung tissue at day 7 p.i. (Figure 3B), also higher alveolitis scores were determined for the NL/602 inoculated animals at day 7 p.i. (Figure 4D).

The bronchial and bronchiolar epithelium from ferrets in both groups showed slight multifocal necrosis with moderate intra-epithelial infiltrates of neutrophils and multifocal peribronchiolar infiltration of macrophages, lymphocytes, neutrophils and plasma cells. The lumina contained moderate amounts of mucus mixed with cellular debris and few neutrophils. The tracheal epithelium in both groups showed mild neutrophilic infiltrates. The severity of both bronchiolitis and tracheitis increased from day 4 to 7 p.i. in ferrets infected with both viruses, but the differences in scores between groups were minimal (Figure 4E and F).

### I223R harboring isolate is transmissible via aerosols or respiratory droplets

Individually housed ferrets were inoculated with virus isolate NL/2631-R223 or NL/602 and naïve animals were placed in a cage adjacent to each inoculated ferret at day 1 p.i. to allow aerosol or respiratory droplet transmission. All inoculated ferrets started to shed virus at day 1 p.i. with virus titers up to 10^6^ TCID50/ml in throat and nose swabs (Figure 4A and C).

The naïve ferrets became infected, because of aerosol or respiratory droplet transmission, 1, 2 or 3 days p.e. In the naïve animals, virus was detected in 4 (NL/602), or 3 (NL/2631-R223) out of 4 animals (Figure 4B and D). The exposed animal in the NL/2631-R223 transmission experiment, from which no virus could be isolated, did not seroconvert in the course of the experiment. At day 5 p.e., the presence of the I223R mutation was confirmed by sequencing the NA gene of virus isolated from the throat swabs of the positive animals.

### I223R mutant transmits as well as parental reference virus

**Figure 5:**
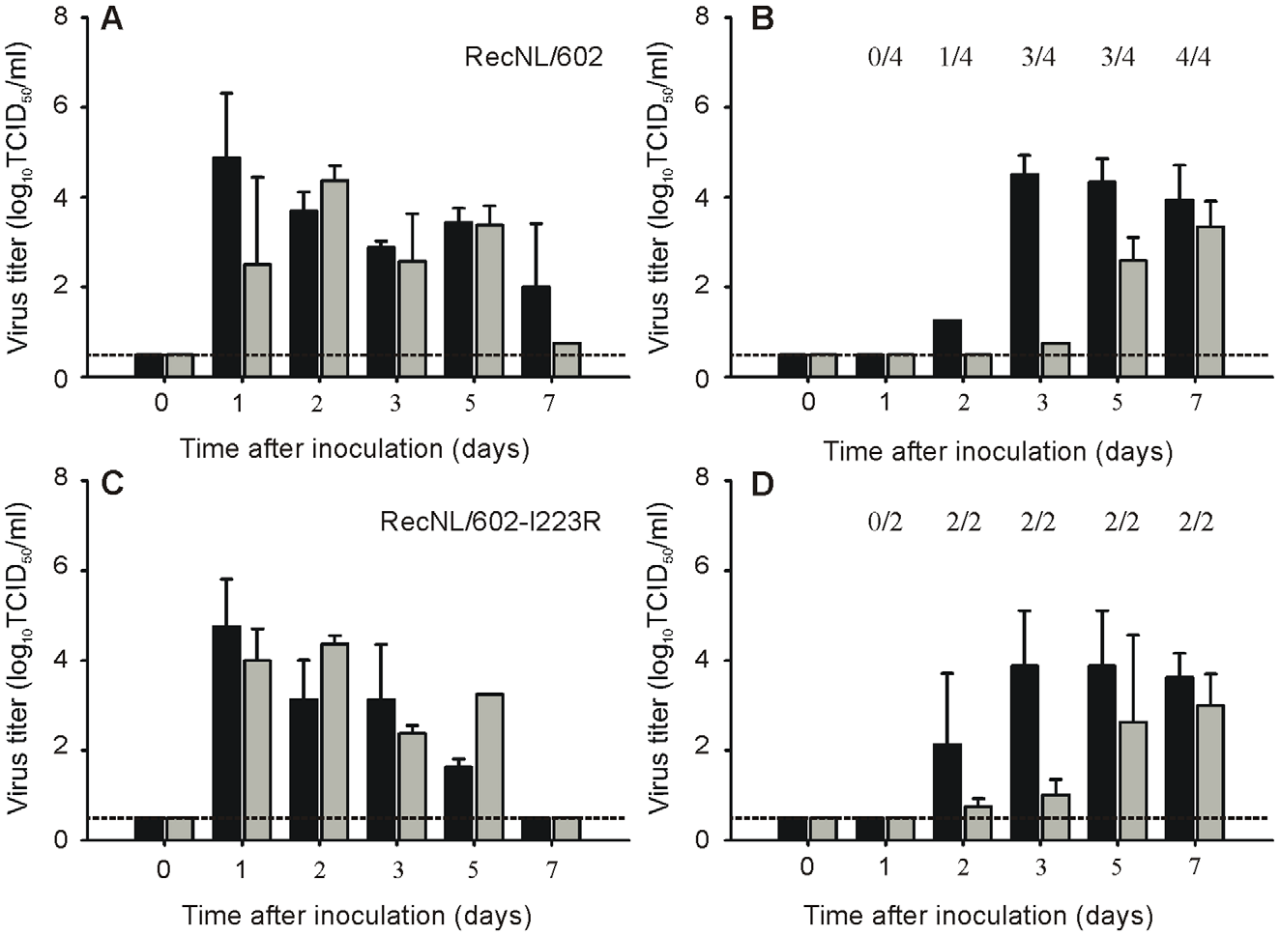
Transmission of recNL/602 and recNL/602-I223R by aerosol or respiratory droplets in ferrets. Virus titers in throat (black bars) and nose swabs (grey bars) are displayed for inoculated (Panel A and C) and exposed ferrets (Panel B and D). The geometric mean titers of positive samples are displayed and the error bars indicate the standard deviations. The number of positive exposed animals per day is depicted. The lower limit of detection is indicated by the dotted line.

When the multi-cycle replication kinetics were studied of viruses with or without the I223R substitution in MDCK cells, it was noticed that the recombinant virus in which the I223R mutation was introduced, recNL/602-I223R, replicated to lower titers than its parental virus recNL/602 (Figure 1). To address if this difference in *in vitro* replication capacity could be extrapolated to reduced replication *in vivo*, the ability of recNL/602-I223R to transmit in the ferret model was studied. It was expected that reduced replication in ferrets would impede the virus to transmit to naïve animals, thereby suggesting that compensatory mutations are needed to balance the fitness loss induced by the I223R mutation. In contrast to the results obtained in MDCK cells, recNL/602-I223R replicated and transmitted as well as recNL/602 when evaluated in the ferret transmission model. Inoculated animals started to shed virus from the upper respiratory tract from day 1 p.i. onwards and transmission was detected in 4 out of 4 (recNL/602), or 2 out of 2 (recNL/602-I223R) naïve animals from day 2 onwards (Figure 5). The presence of the I223R mutation in the recNL/602 backbone was confirmed in throat samples obtained from these animals at day 5 p.e.

## Discussion

Here, a 2009 pandemic influenza A/H1N1 virus isolate, harboring an I223R multidrug resistance mutation, was characterized by studying its replication capacity in MDCK cells and its pathogenicity and transmissibility in the ferret model. This I223R mutant virus is not attenuated for replication in the ferret respiratory tract and transmitted as well as NAI susceptible reference virus NL/602. Furthermore, it was demonstrated here that compensatory mutations for the I223R mutation are not required, since recombinant NL/602 with a single I223R change transmitted as efficiently as its parental virus in ferrets.

To date, 2009 pandemic viruses with an amino acid substitution at position 223 have only sporadically been isolated from patients. A I223V/H275Y double mutant was detected in two closely residing patients who were treated with OS [30]. Besides the I223R single mutant virus studied here, an I223R/H275Y double mutant was detected in an immune suppressed patient treated with OS and ZA [26]. The combination of these mutations resulted in an increased NAI resistance pattern, as compared to the resistance induced by the single mutations. This emphasizes that neuraminidase position 223 is an important marker for antiviral resistance and may be a key residue in the emergence of influenza viruses with resistance to all NAIs, especially in combination with other resistance-associated mutations. So far, the incidence of 2009 pandemic viruses with a 223 change is very low. Notably, 2009 pandemic viruses were reported with a serine to asparagine change at position 247 [31]. In combination with the H275Y change, these viruses demonstrated resistance patterns similar to the I223R/H275Y mutant.

In a pathogenesis experiment, no statistical significant differences were found when weight loss was compared of ferrets inoculated with clinical isolates NL/2631-R223 or NL/602 (Figure 2A and B). In agreement with high viral loads found in respiratory specimens collected from the patient who was infected with NL/2631-R223, high viral loads were detected in the throat of animals inoculated with the same virus. Overall, identical patterns of virus shedding were observed during the course of the experiment in the throats of animals inoculated with either virus. However, virus shedding from the nose could not be detected in all inoculated animals. Although virus shedding from the nose of NL/2631-R223-inoculated animals seem somewhat delayed in comparison with NL/602-inoculated animals, these differences were not significant due to the large variations within groups and small group size after day 4 p.i. (Figure 2C and D).

Both macroscopic and microscopic evaluation of the lungs of the ferrets at day 4 p.i., revealed no major differences in the percentage of affected lung tissue and relative lung weights between NL/2631-R223 and NL/602 (Figure 3B and C). However, at day 7 p.i. the lungs of ferrets inoculated with NL/2631-R223 had not further deteriorated, whereas the percentage of affected lung tissue had increased to 50% in the NL/602 inoculated animals (Figure 3B). This higher score for affected lung tissue in the NL/602-inoculated animals was also reflected by the higher score for the degree of alveolitis at day 7 p.i. compared to day 4 p.i., whereas the alveolitis scores in the NL/2631-R223-inoculated animals at day 4 and 7 p.i. were similar. To recapitulate, both viruses replicated to the same extent in the respiratory tract of ferrets, but the NL/2631-R223 seemed less pathogenic compared to the NL/602 virus.

Despite the moderate pathogenicity of NL/2631-R223, this virus transmitted to 3 out of 4 exposed animals via aerosols or respiratory droplets (Figure 4B). This result is comparable to the data obtained from NL/602, in which 4 out of 4 exposed animals got infected (Figure 4D) [27]. This ferret transmission model was designed as a qualitative model for transmission and with the limited number of animals, quantitative information on virus transmission could not be obtained. Therefore, from these experiments it was concluded that both NL/2631-R223 and NL/602 transmitted via aerosols or respiratory droplets, although a delay in virus shedding by approximately 1 day was observed in the naïve animals exposed to NL/2631-R223 (Figure 4B and D).

When the impact of the single I223R mutation in the recombinant NL/602 backbone on *in vitro* replication kinetics was evaluated, a reduction in virus replication in MDCK cells was noticed (Figure 1). In addition, the initial virus replication of NL/602-I223R on MDCK-SIAT1 cells started 6 to 12 hours later as compared to its parental virus (Figure 1B).

These results suggested that compensatory mutations may be required to accommodate the isoleucine to arginine substitution at position 223 in NA and emphasizes the importance of the viral backbone used to study resistance-associated mutations. However, when recNL/602-I223R was tested in the ferret transmission model, the virus transmitted to 2 out of 2 exposed animals (Figure 5B). When these results were compared with transmission data of recNL/602 (Figure 5D) [32], no differences were found in the onset of virus shedding and virus titers that were detected in the collected throat and nose swabs from the exposed animals. This observation demonstrates that the transmissibility of recNL/602-I223R is not significantly diminished or can at least not be studied using a ferret transmission model.

Although these results suggest that introduction of the I223R does not attenuate the virus, it cannot be ruled out that other mutations than 223R in NL/2631-R223 may have compensated for the initial loss of fitness due to the I223R mutation. Sequence comparison revealed 5 amino acid differences between NL/2631-R223 and NL/602. The only amino acid substitution that is located near the active site of the neuraminidase is at position 248, where NL/602 harbors an aspartic acid and NL/2631-R223 an asparagine. Interestingly, neighboring residue 247 has been linked to NAI resistance in combination with the H275Y mutation [31]. Further research is needed to study the I223R resistance mechanism in competitive mixture experiments and potential co-mutations on a molecular level [33].

To note, small differences between NL/602 and recNL/602 could be observed in replication capacity and transmission patterns in ferrets (Figure 4 and 5). Previously, differences were also found in pathogenesis experiments, where the wild type NL/602 was detected more abundantly in the lower airways of ferrets than recNL/602 [34]. These observed differences may be a result of the use of a virus isolate rather than a virus generated by reverse genetics and to a different batch of ferrets used in the different studies. A direct comparison between virus isolates and recombinant viruses can, therefore, not be made.

The different inoculation routes and inoculation doses used for influenza research is subject of debate. The intratracheal route of inoculation is often used to study pathogenicity or to study the efficacy of vaccines to prevent lower respiratory tract infection. In contrast, the intranasal route of inoculation is used when transmissibility is studied. Unfortunately, these inoculation routes and inoculation doses do not accurately mimic the natural way of infection and may mask the fitness differences between the drug-resistant and drug sensitive viruses.

However, the recipient animals in the transmission experiment are infected via the natural route; aerosols or respiratory droplets shed by the donor ferret. The virus secretion pattern, which is the combination of the amount of virus secreted and the duration of virus shedding from the upper respiratory tract, of animals exposed to recNL/602 and rec/NL602-I223R are similar. This suggests that no marked differences in viral fitness are introduced by the single I223R mutation.

The present study demonstrates for the first time that a 2009 pandemic A/H1N1 clinical isolate containing a resistance mutation at position 223 in the NA is not attenuated in its replication capacity and transmissibility in a ferret model. Although the pathogenicity of this virus seems less severe compared to a relevant reference virus in the ferret model, it is unclear whether this moderate pathogenicity has implications for infections with multidrug-resistant viruses in humans. Continuous surveillance is needed to monitor the emergence of (novel) influenza viruses with reduced susceptibility to the NAIs or mutations that may facilitate the emergence of circulating multi drug resistant influenza viruses.

## Materials and Methods

### Ethics statement

Animals were housed and experiments were conducted in strict compliance with European guidelines (EU directive on animal testing 86/609/EEC) and Dutch legislation (Experiments on Animals Act, 1997). All animal experiments were approved by the independent animal experimentation ethical review committee ‘stichting DEC consult’ (Erasmus MC permit number EUR1821) and were performed under animal biosafety level 3+ conditions. Animal welfare was observed on a daily basis, and all animal handling was performed under light anesthesia using ketamine to minimize animal suffering. Influenza virus seronegative 6-month-old female ferrets (*Mustella putorius furo*), weighing 800–1000 g., were obtained from a commercial breeder.

### Cells and viruses

Madin-Darby Canine Kidney (MDCK) cells were obtained from American Type Culture Collection. MDCK-SIAT1 cells, constitutively expressing the human 2,6-sialyltransferase (SIAT1), were kindly provided by Professor H.D. Klenk, Philipps University Marburg [35]. Both cell lines were cultured in Eagle’s minimal essential medium (EMEM) (Lonza, Breda, The Netherlands) supplemented with 10% fetal calf serum (FCS), 100 IU/ml penicillin, 100 µg/ml streptomycin, 2mM glutamine, 1.5mg/ml sodium bicarbonate (Cambrex), 10 mM HEPES (Lonza) and non-essential amino acids (MP Biomedicals Europe, Illkirch, France). In addition, MDCK-SIAT1 cells were cultured in the presence of 1 mg of antibiotic G418/ml. Influenza virus A/Netherlands/2631_1202/2010 (NL/2631-R223) was isolated from a 5-year-old immune compromised child [25]. Clonal virus of this isolate was obtained by passaging this virus 3 times under limiting diluting conditions in MDCK cells. Full genome sequencing after the last MDCK passage confirmed the absence of mutations. Influenza A/Netherlands/602/2009 (NL/602) was characterized previously [27]. All eight segments of this virus were cloned in a bidirectional reverse genetics plasmid pHW2000 and used to generate recombinant viruses by reverse genetics as described previously [36]. The I223R mutation was introduced in the NA gene of NL/602 using QuickChange multi site-directed mutagenesis kit (Stratagene, Leusden, The Netherlands) resulting in recombinant viruses recNL/602-I223R. The presence of this mutation was confirmed by sequencing.

### Virus titrations

Virus titers in nasal and throat swabs, homogenized tissue samples, or samples for replication curves were determined by endpoint titration in MDCK cells. MDCK cells were inoculated with 10-fold serial dilutions of each sample, washed 1 hour after inoculation with phosphate-buffered saline (PBS), and grown in 200 µl of infection medium, consisting of EMEM supplemented with 100 U/ml penicillin, 100 µg/ml streptomycin, 2 mM glutamine, 1.5 mg/ml sodium bicarbonate, 10 mM HEPES, nonessential amino acids, and 20 µg/ml trypsin (Lonza). Three days after inoculation, the supernatants of inoculated cell cultures were tested for agglutinating activity using turkey erythrocytes as an indicator of virus replication in the cells. Infectious-virus titers were calculated from 4 replicates by the method of Spearman-Kärber [37].

### Replication curves

Multi-cycle replication curves were generated by inoculating MDCK or MDCK-SIAT1 cells at a multiplicity of infection (MOI) of 0.001 50% tissue culture infectious dose (TCID_50_) per cell. One hour after inoculation, at time point 0, the cells were washed once with PBS, and fresh infection medium was added. The supernatants were sampled at 6, 12, 24, and 48 h post infection and the virus titers in these supernatants were determined by means of endpoint titration in MDCK cells.

### Animal experiments

#### Pathogenesis

The pathogenesis experiment was done as described previously with some minor changes in the protocol [29]. On day 0, the ferrets were inoculated intratracheally with 10^6^ TCID_50_ of NL/602 or NL/2631-R223. Throat and nose swabs were collected daily to determine virus excretion from the upper respiratory tract. Animals were weighted daily as indicator of disease and observed for clinical signs. Three animals from each group were euthanized and necropsied at days 4 and 7, and trachea and lung samples were collected to study virus distribution.

#### Pathology

Necropsy was done by opening the thoracic and abdominal cavities and examining all major organs. Whilst inflated, all lung lobes (left cranial lobe, left caudal lobe, right cranial-, middle- and caudal lobes and accessory lobe) were evaluated. The extent of consolidation was estimated by visual assessment. The lungs were weighed after the trachea was removed at its bifurcation. The relative lung weights were calculated as proportion of the body weight on day of death (lung weight/body weight x 100). Tissues (∼0.4 g) from the right lung were collected for determination of lung virus titers at day 4 and 7 p.i. The left lung and trachea were collected for histological examination, and immersed for fixation in 10% neutral-buffered formalin. All samples were sectioned in a standardized way (a total of 4 lung sections per animal; 1 cross section and 1 longitudinal section from both the left cranial and left caudal lobe, and 1 central tracheal cross section) and routinely processed, paraffin embedded and cut to 4 µm hematoxylin and eosin (H&E) stained slides. The samples were histologically examined for the character and severity of influenza virus–associated lesions without knowledge of the identity of the animals. The extent of alveolitis/alveolar damage (0 = 0%, 1 = <25%, 2 = 25-50%, 3 = >50% of a section) and the severity of alveolitis, bronchi(oli)tis (including bronchial submucosal glands) and tracheitis (0  =  none, 1  =  few, 2  =  moderate number, 3  =  many inflammatory cells) were scored per slide. The overall histology score for alveolitis is the sum of the scores for the extent and severity of the alveolitis (score 0 to 6).

#### Transmission

The transmission experiments were done as described previously [27]. The transmission cages were specifically designed to allow transmission experiments to be conducted in negatively pressurized isolator cages (1.6 m × 1 m × 1 m). On day 0, 4 or 2 female ferrets were housed individually in transmission cages (30 cm × 30 cm × 55 cm, W x H x L) and inoculated intranasally with 10^6^ TCID_50_ of NL/602, NL/2631-R223, recNL/602 or recNL/602-I223R respectively, divided over both nostrils (2×250 µl). On day 1, 4 or 2 naïve female ferrets were individually placed in a transmission cage adjacent to an inoculated ferret, separated by two stainless steel grids. Negative pressure within the isolator cage is used to direct a modest (<0.1 m/sec) flow of high efficiency particulate air (HEPA) filtered air from the inoculated to the naive ferret. This experimental setup was designed to prevent direct contact or fomite transmission, but to allow airflow, thereby permitting transmission via aerosol or respiratory droplets. Nasal and throat swabs were collected on day 0, 1, 2, 3, 5 and p.i. from the inoculated ferrets and on days 0, 1, 2, 3, 5 and 7 p.e. from the naïve ferrets. Inoculated ferrets were euthanized at day 7 p.i., and naive ferrets that were found positive by reverse transcription polymerase chain reaction at day 7 p.e. were also euthanized [38]. Naïve animals that remained negative for virus excretion throughout the experiment were euthanized at day 15 p.e., and a blood sample was collected for serology. Virus titers in the collected swabs were determined by means of endpoint titration in MDCK cells.

### Statistical analysis

For the pathogenesis experiment, statistical analysis was done for each time point, until 4 days after inoculation (when there were still 6 animals present in each group). The Mann-Whitney-U test was used to compare weight losses and virus shedding of the six animals in both groups. *P*-values less than 0.05 were considered significant.
